# Suprascapular notch morphology in the pediatric population: a computed tomography study

**DOI:** 10.1007/s12565-016-0364-8

**Published:** 2016-08-18

**Authors:** M. Podgórski, M. Polguj, M. Topol, A. Kusak, M. Łukaszewski, P. Grzelak

**Affiliations:** 1 Department of Diagnostic Imaging, Polish Mother’s Memorial Hospital Research Institute, ul. Rzgowska 281/289, 93-338 Lodz, Poland; 2 Department of Angiology, Interfaculty Chair of Anatomy and Histology, Medical University of Lodz, Lodz, Poland; 3 Department of Normal and Clinical Anatomy, Interfaculty Chair of Anatomy and Histology, Medical University of Lodz, Lodz, Poland; 4 Department of Radiology and Diagnostic Imaging, Medical University of Lodz, Lodz, Poland

**Keywords:** Computed tomography, Children, Development, Bone maturation

## Abstract

Suprascapular notch is characterized by variable morphology. However, its development is not well studied. We hypothesize that it proceeds postnatally. Thus, the aim of this research was to characterize the morphology of the suprascapular notch in a pediatric population based on computed tomography. A retrospective analysis was performed of 291 chest computed tomography examinations of patients under 18 years old taken following other clinical indications. The inclusion criteria were as follows: both scapulae encompassed in a field of view; no artifacts; no pathologies concerning the scapulae. Based on visual assessment and measurements, the suprascapular notch was classified according to a fivefold classification (type I, deeper than wider; type II, equally deep and wide; type III, wider than deeper; type IV, bony foramen; type V, discreet notch). In all, 173 examinations were included (60 females and 113 males). The most common suprascapular notch types were discreet notch (type V, 225 scapulae; 65.0 %) and type III (114 scapulae; 32.9 %). Children with type V suprascapular notch were significantly younger than children with other types (26.1 ± 42.4 months vs. 111.2 ± 66.7 months; *p* < 0.05). In types I–III, a positive correlation was found between age and dimensions of the suprascapular notch (*p* < 0.05). This study provides the first description of the suprascapular notch in a pediatric population based on computed tomography. It confirms that morphology of the suprascapular notch undergoes postnatal development.

## Introduction

The suprascapular notch (SSN) is a hollow located on the upper edge of the scapula, at the base of the coracoid process. It holds the suprascapular nerve, which is enclosed in the SSN by the superior transverse scapular ligament, attaching to the upper border of the SSN. The SSN is the most common site of suprascapular nerve injury. Entrapment of this nerve can create pain symptoms of the shoulder region; however, it may also cause inappropriate stimulation of the supraspinatus and infraspinatus muscles, resulting in their atrophy and eventual reduction of the abduction and external rotation of the upper limb (Zehetgruber et al. 2002).

The morphology of the SSN is highly variable, and some of these variants (e.g., narrow and deep SSN) may predispose the patients to suprascapular nerve injury (Natsis et al. 2007). Hence, this region has been well explored based on cadaveric studies. Moreover, recent radiological studies employing computed tomography (CT), magnetic resonance imaging and ultrasound have assessed anatomical variations of the SSN (Inoue et al. 2014; Simeone et al. 2015; Polguj et al. 2013a).

However, the aforementioned studies concern only adults and do not contain any data on the appearance of the SSN in the pediatric population or on its developmental changes. Due to the fact that CT examination exposes the patient to high doses of radiation, its use cannot be justified only for the evaluation of SSN morphology, especially in children. However, the SSN region may be visualized in the background of a chest CT examination performed in response to other medical indications.

This article examines the morphological variations of the suprascapular notch in a pediatric population based on computed tomography. To our knowledge, such a study has never been published before.

## Materials and methods

The study was based on a retrospective analysis of 291 single-phase (arteria), 128-row, helical computed topography examinations of the chest. The inclusion criteria comprised age under 18 years old and visualization of the whole scapulae on both sides. Exclusion criteria were congenital skeletal defects of the shoulder region and posttraumatic changes concerning the scapulae. The study protocol was approved by the local bioethics committee (protocol no. 28/2015) and was performed in accordance with the ethical standards laid down in the Declaration of Helsinki.

Examinations were performed with a The Brilliance iCT SP CT scanner (Philips, The Netherlands; slice thickness 0.5–1 mm, kVp 80, mAs 50–100, iDose 6) between November 2011 and July 2015 because of other clinical indications. Patients were positioned prone with their hands above the head to minimize the radiation dose.

Shoulders were analyzed with post-processing tools incorporated into the IntelliSpace Portal 6.0 (Philips, The Netherlands) obtaining multiplanar reconstruction images along the coronal and sagittal planes and with three-dimensional volume-rendering reconstructions of the scapulae. Measurements were performed from the anterior side of the scapula, with the suprascapular notch parallel to the coronal plane. They were obtained by a single radiologist with 10 years of experience in evaluation of tomographic examinations. Additionally, to evaluate intra- and interrater agreement, 50 randomly selected examinations were reevaluated by the same radiologist and by the second radiologist with 7 years of experience in evaluation of tomographic examinations, respectively.

**Fig. 1:**
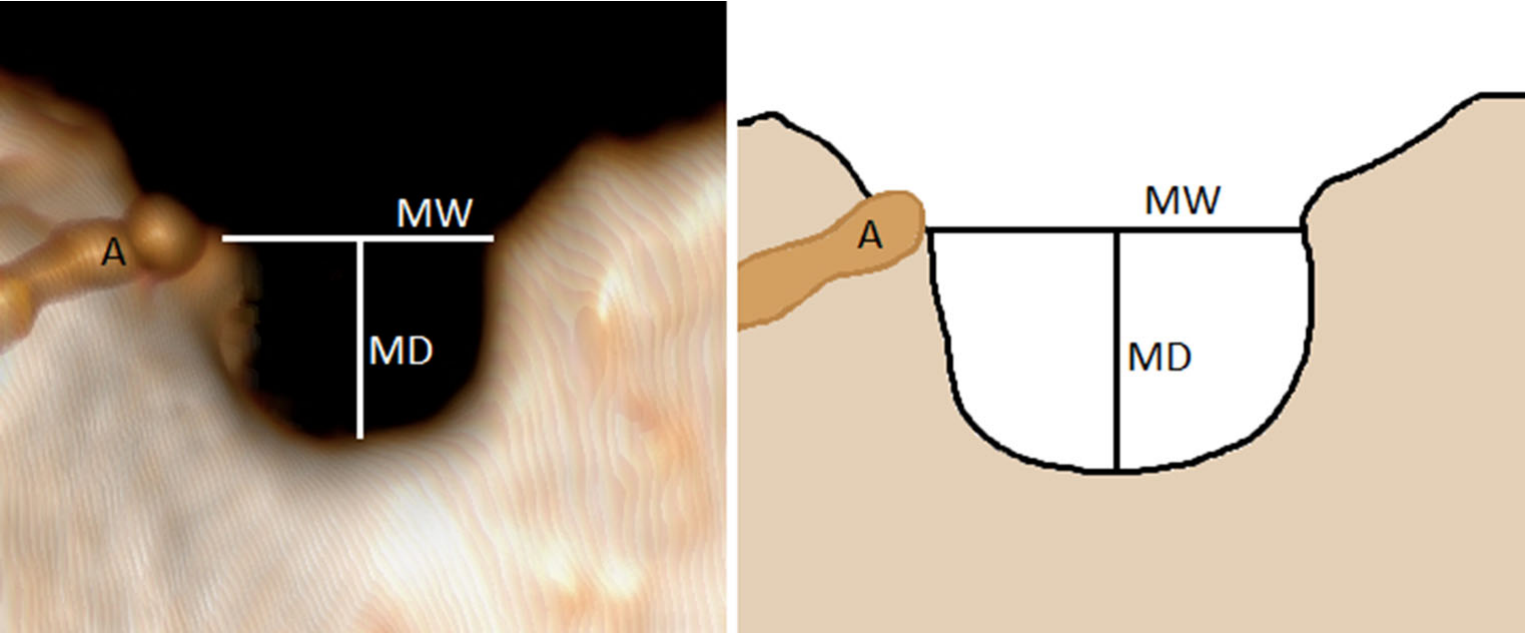
Measurements of the suprascapular notch presented on a computed tomography 3D reconstruction (**a**) and a parallel schematic drawing (**b**). *MW* maximal width, *MD* maximal depth, *A* suprascapular artery

The suprascapular notches were classified according to Polguj et al. (2011). In this classification, types IV (suprascapular opening) and V (discreet notch) are recognized based on visual assessment. Types I–III were assigned based on measurements of maximal width (MW: distance between the most distant points of the upper edge of the SSN) and maximal depth of the SSN (MD: imaginary line between the level of the MW and the deepest point of the SSN) (Fig. 1). Maximal width (MW) is 0.5 mm less than the maximal depth (MD) in type I (MW < MD), equal to MD in type II (MW = MD), and 0.5 mm greater than MD in type III (MW > MD).

Statistical analysis was performed using Statistica 12 software (StatSoft Poland, Cracow, Poland), and a *p* level of <0.05 was considered significant. Continuous variables are presented as mean and standard deviation and nominal variables as a number and percentage. The normality of the data distribution was checked with the Shapiro-Wilk test. The Mann-Whitney test was used to compare continuous variables between two groups, and the Wilcoxon sign-rank test was used to compare two parameters within the same patient. To evaluate intra- and interrater agreement, the kappa statistic was used for classification of the SSN type and Pearson’s correlation coefficient for measurement of the MD.

## Results

In total, 173 CT scans were included in the study (346 scapulae): within this group, 60 patients were females (aged 5.7 ± 7.5 years) and 113 were males (aged 4.3 ± 5.3 years). No significant age difference was found between genders; 118 CT scans were excluded from analysis because the field of view did not encompass the whole scapula on both sides.

**Fig. 2:**
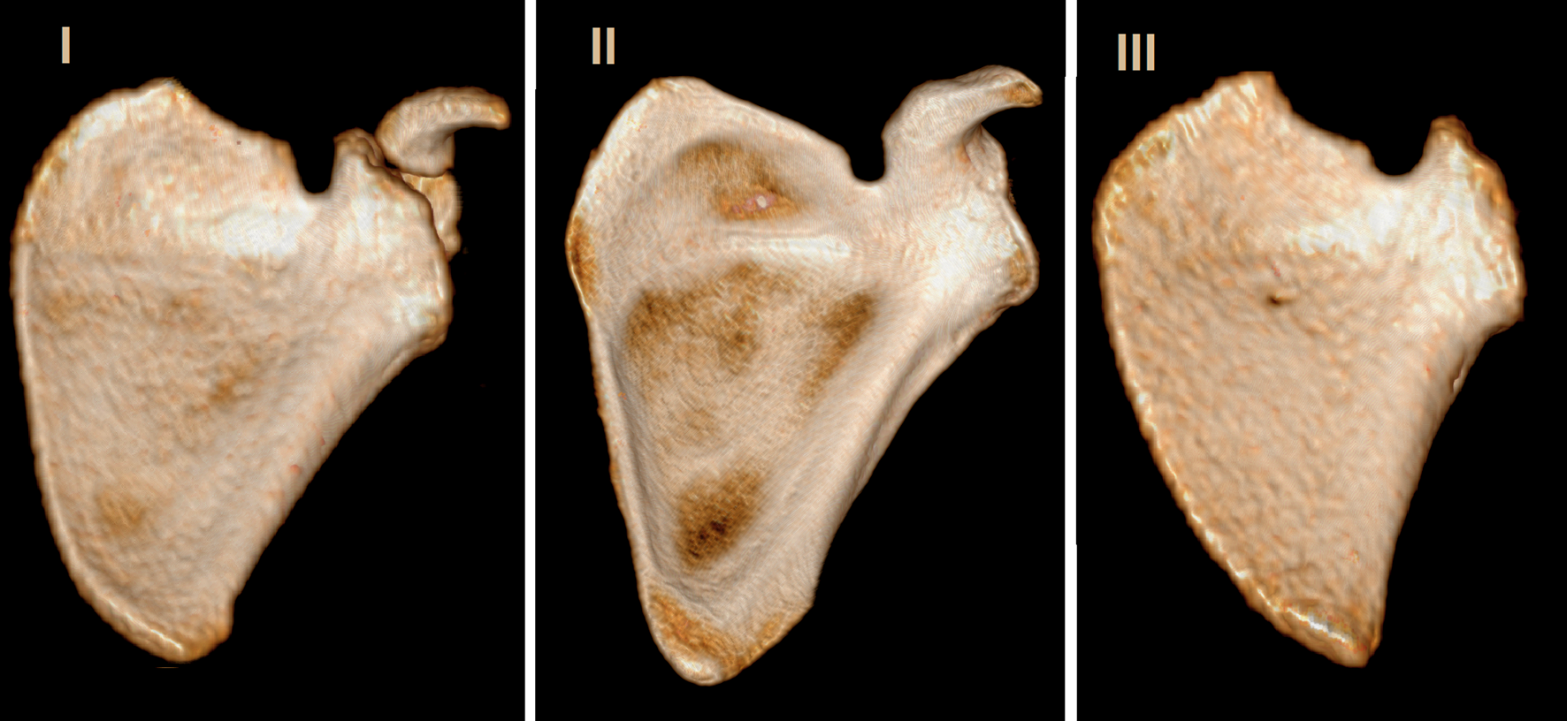
Three-dimensional reconstruction of the scapula with types I–III of the suprascapular notch

Among all 346 scapulae, type V (in 225 scapulae; 65.0 %) and type III (in 114 scapulae; 32.9 %) SSNs were most common. Types I and II were present in 5 (1.4 %) and 2 (0.6 %) scapulae, respectively. Figure 2 shows the representative cases of SSN types I–III.

**Fig. 3:**
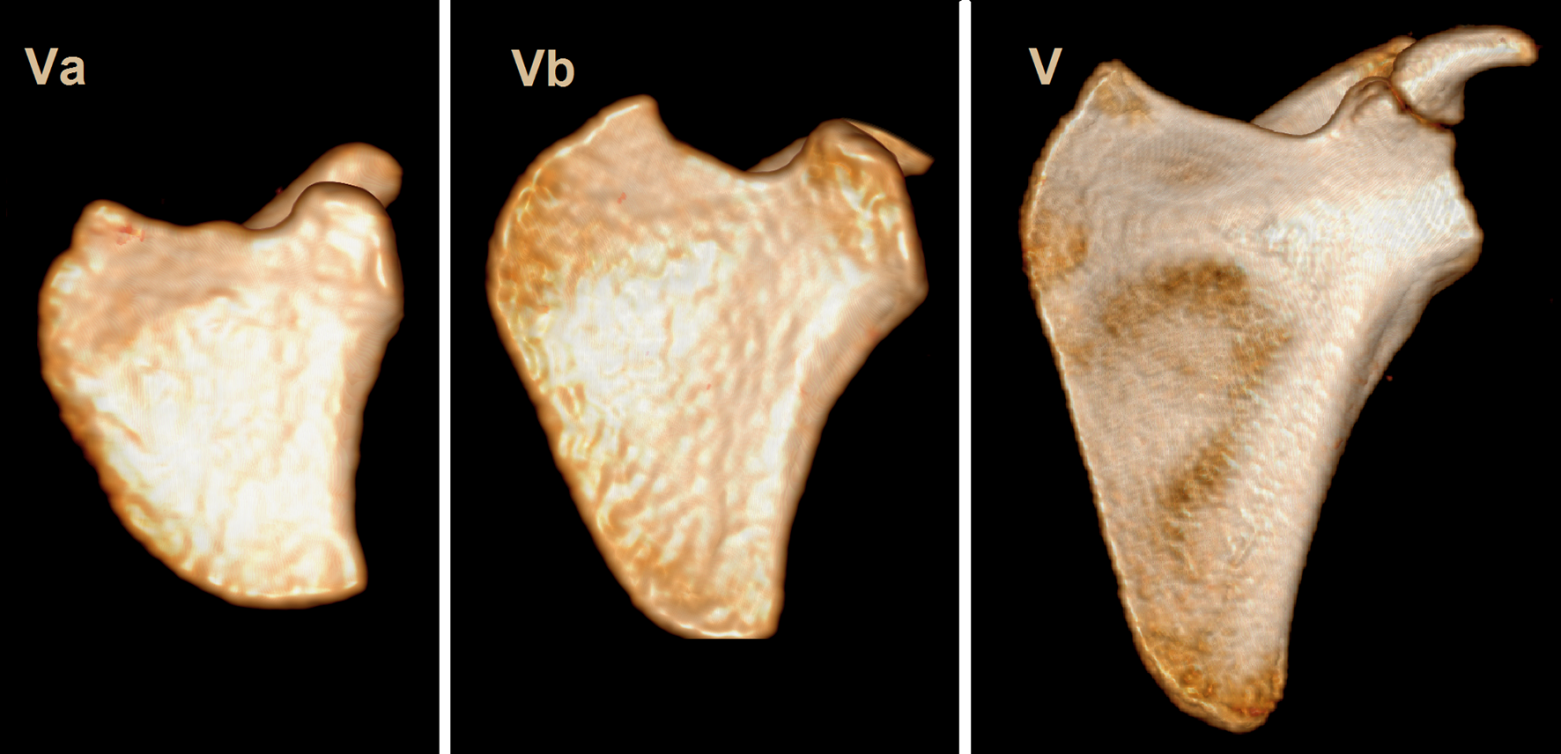
Three-dimensional reconstruction of the scapula with types Va, Vb and matured type V of the suprascapular notch

No scapulae were found with a type IV SSN (bony foramen). The distribution of notch types according to body sides and gender is presented in Table 1. Of the 225 scapulae presenting a type V SSN, 16 cases also presented a tubercle on the superior border of the scapula, medially to the base of the coracoid process (Fig. 3).

**Table 1:** Distribution of suprascapular notch types in the whole group and within each gender

Notch type	General (*n* = 173) [number (%)]		Female (*n* = 60) [number (%)]		Male (*n* = 113) [number (%)]	
	Right	Left	Right	Left	Right	Left
I	2 (1.2)	3 (1.7)	1 (1.7)	2 (3.4)	1 (0.1)	1 (0.1)
II	2 (1.2)	0	1 (1.7)	0	1 (0.1)	0
III	54 (31.2)	60 (34.7)	23 (38.3)	24 (40.0)	31 (27.4)	36 (31.9)
IV	0	0	0	0	0	0
V	115 (66.5)	110 (63.6)	35 (58.3)	34 (56.6)	80 (70.8)	76 (67.3)

The dimensions of SSN types I–III are presented in Table 2. No significant difference in MW and MD was observed between sides of the body (MW 10.9 ± 3.5 mm on the right side vs. 11.0 ± 3.3 mm on the left side, *p* = 0.8748; MD 5.3 ± 2.7 mm on the right side vs. 5.3 ± 2.3 mm on the left side, *p* = 0.8420).

**Table 2:** Dimensions of suprascapular notches in the whole group and within each gender

SSN type		Whole group [mm (SD)]		*p*	Female [mm (SD)]		*p*	Male [mm (SD)]		*p*
		Right	Left		Right	Left		Right	Left	
MW	I	6.9 (0.3)	8.2 (2.1)	0.773	7.1	9.4 (0.3)	–	6.7	5.7	–
	II	7.7 (3.3)	–	–	10.0	–	–	5.4	–	–
	III	11.2 (3.4)	11.2 (3.3)	0.970	11.3 (3.5)	11.3 (3.2)	0.999	11.2 (3.4)	11.1 (3.4)	0.97
MD	I	8.2 (0.4)	9.8 (3.3)	0.539	8.4	11.7 (0.6)	–	7.9	6.1	–
	II	7.2 (4.0)	–	–	10.0	–	–	4.3	–	–
	III	5.2 (2.6)	5.0 (2.0)	0.713	4.4 (2.3)	4.6 (2.0)	0.713	5.8 (2.8)	5.2 (2.0)	0.39

*SSN* suprascapular notch, *MW* maximal width, *MD* maximal depth, *SD* standard deviation

**Fig. 4:**
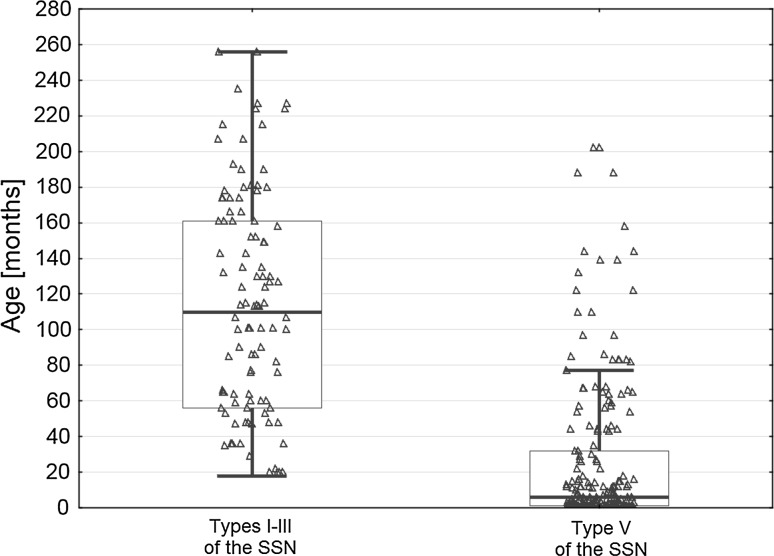
Difference in age between children with type V of the SSN and all other types of the SSN taken together. Thick *horizontal line* represents mean, *box* shows standard error, *whiskers* indicate minimum and maximum values, and *triangles* correspond to row data

**Fig. 5:**
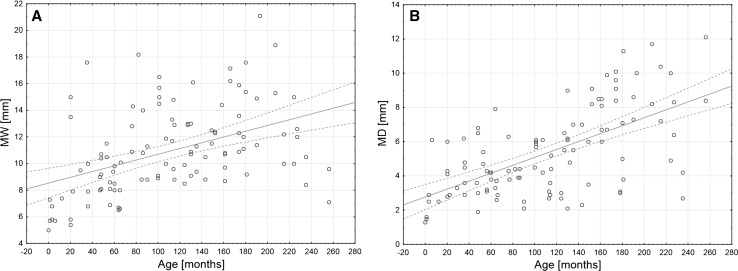
Scatter plots of correlation between age and maximal width of the suprascapular notch (**a**) and maximal depth of the suprascapular notch (**b**)

Due to the fact that there were only five notches of type I and two of type II, a comparison was made of the mean age of the patients with type V SSN and those with all other types combined. Children with type V SSN were significantly younger than children with other SSN types (26.1 ± 42.4 months vs. 111.2 ± 66.7 months, respectively; *p* < 0.05) (Fig. 4). Moreover, in types I–III, there was a positive correlation between age and MW (*R* ^2^ = 0.4368; *p* < 0.05) and with MD (*R* ^2^ = 0.6167; *p* < 0.05) (Fig. 5).

Cohen’s kappas for intra- and interrater agreement were 0.96 (CI 0.89–1) and 0.92 (CI 0.81–1), respectively. Pearson’s correlation coefficients for intra- and interrater agreement were 0.85 and 0.81 (*p* < 0.05).

## Discussion

Diagnostic imaging has become a useful tool in anatomical studies. In a pediatric population, magnetic resonance imaging and ultrasound are methods of choice because of the lack of exposure to harmful radiation. Moreover, magnetic resonance imaging enables clear visualization of non-ossified cartilaginous epiphyses, ossification centers, and physes (Kwong et al. 2014). Studies concerning the shoulder joint region in pediatric populations using this technique have described the normal skeletal maturation of the proximal humerus (Kwong et al. 2014) and the glenoid and glenoid-coracoid interface (Kothary et al. 2014). The general development of the scapula has been assessed more extensively based on cadaveric studies (Schaefer et al. 2009).

At 12–14 weeks of pregnancy, the morphology of the main body of the scapula is close to that of an adult (Schaefer et al. 2009). At birth, the majority of the main body of the scapula is ossified except for the acromion, coracoid process, medial border, inferior angle, and glenoidal mass, which remain cartilaginous (Schaefer et al. 2009). Ossification progresses up to 23 years of age, when all epiphyses are fused and the adult form is achieved (Schaefer et al. 2009). Although the juvenile scapula morphology has been extensively described, no data describe the shape of the SSN and its development after birth.

According to Graves (1922), age-related morphological changes in the scapula comprise two distinct processes: ossification and atrophy. Ossification concerns cartilaginous structures surrounding the glenoid fossa, clavicular facet, tip of the acromion process, and base of the spine (Cardoso 2008). Atrophy manifests initially as reduction and eventual loss of the surface vascularity as well as the occurrence of atrophy spots (discrete patches of bone atrophy). A more recent study by Dabbs and Moore-Jansen (2012) evaluated age-related changes in a group of 804 adult scapulae derived from an American population. Sex- and ancestry-related developmental changes were identified, which most commonly included increased border thickness, increased ventral curvature (kyphosis), and changes in overall size, such as decreased length and increased breadth of the infraspinous body. The results indicate that changes in the scapula occur not only as a result of variations in age, but also, more importantly, in occupation. Differences in occupation can result in the muscles attached to the scapula undergoing vigorous activity, which may result in bone remodeling. The same process may influence the development of the scapula in a pediatric population and might explain why both the frequency of type V decreases and the depth of the SSN increases with age.

The distribution of the SSN types differs between pediatric patients and adults. This study found type V to be present in 65.0 % of scapulae and type III in 32.9 %. In a study performed on 86 dried scapulae of adults by Polguj et al. (2011), type V was observed in 11.6 % of specimens and type III in 54.7 %. A similar study evaluating the shape of the SNN in chest CTs of adult patients found that among 619 analyzed scapulae, 55.8 % presented type III SSN and 12.9 % type V (Polguj et al. 2013a). A third study based on 762 patients from the Japanese population with a mean age of 58.2 ± 19.1 years (range 10–92 years) used three-dimensional CT to assess the shape of the SSN based on the Rengachary classification (Rengachary et al. 1979). The results indicate that 11.4 % of scapulae were classified as possessing Rengachary type I SSN, which corresponds to our type V. Although previous studies have found this type to be present in 6–21.5 % of cases, these were performed mainly on dried scapulae, and the age of the donors was unknown (Rengachary et al. 1979).

The different SSN type distribution between children and adults and the positive correlation between age and SSN size suggest that the final shape of the SSN develops with age. It should be noticed that pediatric type V SSN presented a more heterogeneous morphology than the adult type V because of the presence of the tubercle on the superior border of the scapula, which was observed in 16 cases. Hence, in children, the type V SSN should be subdivided into types Va and Vb based on the respective absence or presence of this tubercle (Fig. 2). In this sense, type Vb (“type V with tubercle”) may represent a transient form between juvenile type V of the SSN and the adult type of a different kind.

Another argument for the postnatal development of the morphology of the SSN might be the absence of type IV (the suprascapular foramen) in children. The occurrence of this type in adults ranges from 1.92 % (Inoue et al. 2014) to 7.3 % (Polguj et al. 2011). In a Polish population, it was reported in 6.2 % of specimens by cadaveric study and in 5.0 % of cases by CT examination (Polguj et al. 2012; 2013b). In the US population, its frequency is known to range between 3.7 and 5.5 % of cases (Zehetgruber et al. 2002). There is no consensus on the mechanism of its creation. The most common hypothesis assumes that the superior transverse scapular ligament may ossify with age, enclosing the osteofibrosus tunnel into a bony foramen (Inoue et al. 2014). This hypothesis is supported by observations of different degrees of STSL ossification (Polguj et al. 2011). Moreover, Inoue et al. (Inoue et al. 2014) note that patients with a bony foramen were significantly older than patients with other types of SSN. However, the same researchers also report the occurrence of a bony foramen in a 21-year-old patient. Nevertheless, our observations support the sequential development of SSN types I–IV: from type Va, through type Vb, to a final form, creating an osteofibrous tunnel together with the superior transverse scapular ligament and/or anterior coracoscapular ligament (Polguj et al. 2013b), which may further ossify.

A limitation of this study is that the morphology of the SSN was assessed at only one time point. Therefore, the sequence of its development is only speculative. Future studies should include other diagnostic methods for evaluating the SSN in a pediatric population. Comparing ultrasound with CT as a gold standard, Polguj et al. (2015) report good sensitivity and specificity of sonography in differentiating among SSN types I–III. However, this technique did not allow the observer to differentiate between the bony foramen (type IV) and a discreet notch (type V). Nevertheless, our results indicate that this potential source of confusion should not bias the assessment in children because type IV was not found to occur.

The second limitation of this study was performing measurements using 3D reconstructions. The accuracy of morphological assessment of 3D models has been questioned. However, with the development of new tools for data post-processing, the quality of reconstructions has improved enough to perform morphological measurements (Qiang et al. 2014; Jamali et al. 2007) or even to plan surgical procedures (Guo et al. 2015). To confirm the reliability of our calculations, we showed that the intra- and interrater agreement for classification of the SSN type was almost perfect and for direct measurements of MD was strong (McHugh 2012). Thus, in our opinion, it entitles us to draw the following conclusions.

## Conclusion

This is the first study describing the morphology of the SSN in a well-characterized population of pediatric patients. In contrast to adults, certain types of SSN dominate in children, and the suprascapular foramen does not occur at all. Furthermore, the sizes of the suprascapular notch increase with age. All the above suggests that the final shape of the SSN develops postnatally.
